# The effect of the perceived social support on mental health of Chinese college soccer players during the COVID-19 lockdown: The chain mediating role of athlete burnout and hopelessness

**DOI:** 10.3389/fpsyg.2022.1001020

**Published:** 2022-11-11

**Authors:** Liangyu Zhao, Zongyu Liu, Liguo Zhang

**Affiliations:** School of Physical Education, Shandong University, Jinan, Shandong, China

**Keywords:** college soccer players, perceived social support, mental health, athlete burnout, hopelessness

## Abstract

The isolation and blockade during the COVID-19 pandemic have a great impact on the mental health of Chinese college soccer players. This study aimed to explore the effect of perceived social support on mental health of college soccer players, as well as the mediating role of athlete burnout and hopelessness during the COVID-19 lockdown. Based on a sample of 674 college soccer players from several universities in China, the study used the Perceived Social Support Scale (PSSS), Kessler Psychological Distress Scale (K10), Athlete Burnout Questionnaire (ABQ) and Beck Hopelessness Scale (BHS). The results indicated that: (1) there was a significant positive correlation between perceived social support and mental health of college soccer players; (2) the athlete burnout played a mediating role between perceived social support and mental health of college soccer players; (3) the hopelessness also played a mediating role between perceived social support and mental health of college soccer players; (4) the athlete burnout and hopelessness played a chain mediating role between perceived social support and mental health. These findings could contribute to insight into the mechanisms by which perceived social support affects the mental health of college soccer players and provide important practical guidance for protecting and promoting their mental health.

## Introduction

The soccer developed countries all over the world put the youth soccer training on the basis of the soccer cause. In Japan, great attention was paid to the development of youth training of campus soccer, and the dual-ladder promotion pattern of both professional and campus soccer was realized (Cheng and Zhang, 2014). In Germany, amateur soccer clubs promoted soccer in the community, and it took professional clubs as the core of youth training (Liu, 2011). Similarly, the development of youth soccer in China has always been the core topic of soccer reform and development. By 2020, China had selected 201 pilot counties (districts) for campus soccer, 38 pilot areas for campus soccer reform, and 188 colleges and universities recruiting high-level soccer teams (China Youth Daily, 2022). With the development of campus soccer, the number of college soccer players is also increasing. Chinese college soccer players refer to the students who have certain soccer skills and quality and pass the sports training examination of ordinary institutions of higher learning or the recruitment examination of high-level sports teams. At the university level, different from other college students, their main task is to represent the school to participate in provincial and national soccer matches for making contributions to the school and national competitive sports. In addition to the overload of training, they are also exposed to high risk of injury, the uncertainty of their sporting career, and the long-term pressure of school and competition, making them vulnerable to mental health problems (Elbe and Jensen, 2016; Akesdotter et al., 2020). As one of the core elements of athletes, mental health is an important resource for them throughout their career (Henriksen et al., 2019). The World Health Organization (WHO) defined mental health as a healthy state. In this state, each person was able to realize his or her potential, cope with normal life pressures, work productively, and contribute to his or her community (World Health Organization, 2018). The researchers classified athletes’ mental health problems into clinical mental disorders, sub-clinical mental disorders, ordinary people’s situations and athletes’ unique conditions (Henriksen et al., 2019). Sports practitioners should pay more attention to the sub-clinical symptoms of athletes who do not meet the diagnostic criteria for psychological disorders and implement early intervention in a timely manner (Roberts et al., 2016). There were evidences that injuries, stress, burnout and hopelessness that athletes encounter during their sports careers could increase their risk of mental health problems (Abbott et al., 2019; Reardon et al., 2019). The coronavirus disease-2019 (COVID-19) outbreak caused national sports competitions to be suspended, and college soccer players had to train at school or home to maintain skill levels. Being isolated and online training had an important impact on the psychology of soccer players (Schinke et al., 2020; Nassar et al., 2021; Sun et al., 2021). Studies found that athletes tended to suffer from greater pressure and hopelessness (Doherty et al., 2016; Schinke et al., 2018), and hopelessness was the direct cause of mental health problems such as depression and suicide tendency (Beck et al., 1974; Li, 2014). Many studies proposed that a high level of perceived social support had a positive effect on mental health (Fan et al., 2021), which could not only help athletes reduce athlete burnout (Gustafsson et al., 2017), but also reduce pressure, anxiety, helplessness and hopelessness related to competitive sports (Noblet et al., 2003; Lamis et al., 2016). Therefore, it is important to explore the mental health of Chinese college soccer players during the COVID-19 lockdown for their subsequent performance and development.

During the COVID-19 pandemic, social support was an important factor for athletes. Social support usually referred to various forms of support and help provided by an individual’s social network system, such as care, attention or respect from other members of the social network, which directly affect an individual’s mental health (Uchino et al., 1996). The resource protection theory regarded social support as an external supplement of resources, and people could rely on social support to avoid or reduce psychological pain in stressful environments (Hobfoll et al., 2016). Studies found that the link between social support and mental health might be more prominent among adolescents (Heerde and Hemphill, 2018). In youth sports, social support mainly came from coaches, family members, and peers (Freeman and Rees, 2008; Sheridan et al., 2014). As an important external resource, social support could significantly improve the investment level of adolescent athletes, positively predicted their sports performance (Qiu, 2015; Ye et al., 2016), and helped alleviate the psychological pressure of athletes (Johnston and Carroll, 1998). Social support was divided into actual received social support and perceived social support (Uchino, 2009), in which perceived social support referred to the subjective perceived judgment of individuals on available social support (Qiu, 2015). Studies found that in stressful events, the perceived social support was less than the actual support (Dunkel-Schetter and Bennett, 1990), and perceived social support was often more beneficial to mental health than the actual received social support (Qiu, 2015). The stress buffering hypothesis suggested that high levels of perceived social support protected individuals from potential negative effects of stressors, which was associated with better physical and mental health (Cohen and Wills, 1985). Adolescents’ perceived social support, especially from friends, had a positive effect on their mental health in early adulthood (Jakobsen et al., 2020). Studies found that adolescent athletes generally believed that coaches were the most important support force. Positive and effective social support from coaches, parents and peers could promote athletes’ sport performance and positive psychological feelings, but if athletes perceived less and unreasonable support, it would increased their tendency to retire early (Sheridan et al., 2014). For example, if Olympic athletes felt that they received a high level of social support before the competition, including support from family, coaches, teammates and other support personnel, the pressure of competition would be reduced (Fletcher and Sarkar, 2012). Therefore, perceived social support played an important role in athletes’ mental health.

Freudenberger conducted a study on burnout among volunteers in a drug rehabilitation clinic in New York, so he was generally regarded as the founder of this field (Freudenberger, 1974). Later, Maslach proposed that psychological burnout was composed of emotional exhaustion, dehumanization and personal achievement (three-factor model; Maslach and Jackson, 1982). Studies found that the increase of burnout level would increase the perceived stress and depressive symptoms of college students, and had a negative impact on the mental health for them (Gerber et al., 2015). Similarly, high levels of stress, moderate or severe anxiety symptoms, and moderate or severe depressive symptoms were associated with increased of burnout levels in the postgraduate population (Allen et al., 2020). A study using cross-lag panel analysis further showed that the impact of burnout on depressive symptoms was greater than that of depressive symptoms on burnout (Salmela-Aro et al., 2009). Raedeke first proposed the concept of athlete burnout in the field of competitive sports, and defined it as a syndrome characterized by reduced sense of achievement, emotional/physical exhaustion and negative evaluation of sports (Raedeke, 1997). Studies found that when athlete burnout occurred, athletes would be less interested in training, lack of motivation, extremely tired of training, and even experience anxiety and depression symptoms, which negatively affected their mental health (Demirci and Çepikkurt, 2018). In addition, athlete burnout might lead to athletes quitting training and leaving the industry in advance. Therefore, studying the causes of athletes’ psychological burnout is the key to the prevention and treatment of athlete burnout. Through qualitative research on athlete burnout, Liu and Zhang found that athlete burnout was a predictable phenomenon with gradual development that was accompanied by athletes for a long time and determined by a variety of factors (Fanglin and Liwei, 2004). According to the cognitive-affective stress model, athlete burnout was the stress effect caused by the long-term imbalance of environmental requirements and individual resources, and it was the last stage of psychological collapse when athletes suffered from adverse pressure for a long time (Smith, 1986). Under the unique management and training mode of competitive sports in China’s national system, social support was an important social environment resource that athletes get from coaches, families, teammates and organizations, and an important resource for athletes to cope with external pressure (Fisher, 1985). Lack of social support or perception of support was considered to be one of the strongest factors triggering athlete burnout (Cresswell, 2009; DeFreese and Smith, 2014). Rees et al. showed that higher levels of perceived social support (perceived emotion, respect, information and tangible support) in high-level athletes was associated with lower levels of athlete burnout (Rees and Hardy, 2000). Therefore, perceived social support played an important role in preventing and reducing athlete burnout. In conclusion, we believed that athlete burnout might play a mediating role in the relationship between perceived social support and mental health of college soccer players.

Studies on hopelessness in the field of psychology began in the early 20th century. It was first considered as a negative cognitive expectation system about oneself and future life (Scotland, 1969). Beck et al. defined hopelessness as an individual’s negative concept, negative expectation or pessimism about the future, specifically a cognitive schema of negative expectation for the future (Beck et al., 1974). When encountering negative events, if an individual repeatedly thought about the causes and results of negative emotions, the negative schema would be activated and the individual would have a strong sense of helplessness, which would increase the individual’s sense of hopelessness (Ma, 2016). Abramson et al. defined hopelessness as an expectation that highly desired outcomes would not occur, or that highly aversive outcomes would occur, and the expectation that no response in one’s repertoire would change the likelihood of these outcomes occurring (Abramson et al., 1989). Hopelessness was a psychological change in adolescents, and was the perception of negative results of external factors. Hopelessness theory of depression regarded hopelessness as the proximal and sufficient cause of the symptoms of despairing depression. In other words, hopelessness was the direct cause of depression. According to this theory, individuals prone to hopelessness had two characteristics: the expectation that events highly valued by individuals would produce negative results and individuals would do nothing to change these results (Abramson et al., 1989). In Chinese culture, students study was highly valued, so teenagers may show hopelessness for poor academic performance (Pekrun et al., 2009). A study on university students found that despair was associated with life satisfaction, depression and anxiety (Guney et al., 2010). Several studies showed that despair was a significant predictor variable of depression and that elevated levels of despair in individuals could have a serious negative impact on mental health (Baryshnikov et al., 2008; Serafini et al., 2020). Extending to competitive sports, athletes might also express hopelessness over unsatisfactory athletic performance. Lester’s found that athletes faced greater stress and hopelessness, especially elite athletes (Lester, 2015). Because elite athletes received more attention, more negative social comments and more pressure, they were more likely to be injured, fail or overtrain during training and competition. These could lead to mental health problems. Studies found that social support could reduce individuals’ sense of hopelessness, reduce the correlation between hopelessness and depression, and was a protective factor for suicidal ideation. However, students who lacked perceived social support may experience more serious hopelessness, depression and even suicidal thoughts without the influence or intervention of social networks (Lamis et al., 2016). Therefore, we believed that hopelessness might play a mediating role between perceived social support and mental health in college soccer players.

Athlete burnout was a common negative psychological state of athletes, which included physical/emotional exhaustion, diminished personal accomplishment and negative evaluation of sports. Physical/emotional exhaustion was related to high-intensity training and competition. Diminished personal accomplishment meant that athletes could not complete their personal goals or their sports performance level was lower than expected. Negative evaluation of sports referred to losing interest in sports or resenting sports and achievements (Raedeke and Smith, 2001). Burnout was more common in stressful places such as hospitals and schools. Earlier studies shown that burnout was associated with feelings of helplessness and hopelessness (Kahill, 1988). In a study of psychiatric nurses, burnout was found to promote hopelessness (Pompili et al., 2006). Currently, there are few studies on the relationship between athlete burnout and hopelessness. Researchers found that the lack of hope among athletes might be related to athlete burnout (Gustafsson et al., 2010). Raymond et al. found that long-term hopelessness would affect students’ academic performance and mental health by reconstructing depression model of learned hopelessness (Au et al., 2009). Similarly, Long-term hopelessness could also affect athletes performance and led to mental illness (Gustafsson et al., 2010). In view of this, we believed that athlete burnout and hopelessness might play a chain mediating role between perceived social support and mental health of college soccer players.

**Figure 1:**
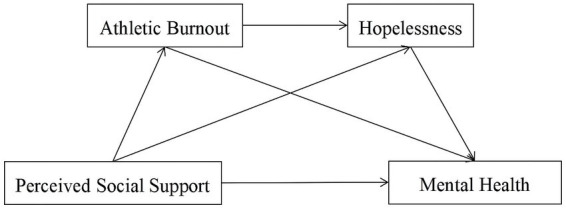
The hypothetical chain mediating effect model of athletic burnout and hopelessness between perceived social support and mental health among college soccer players.

In conclusion, we preliminarily analyzed the relationships among perceived social support, athlete burnout, hopelessness, and mental health. We found that most of the existing studies focus on the relationship between factors, but there were few studies on the mechanism of mental health of college soccer players. Therefore, based on conservation of resources, pressure buffering hypothesis, cognitive-affective stress model and hopelessness theory of depression, we explored the impact of perceived social support on mental health of college soccer players during COVID-19 lockdown, and the mediating role of athlete burnout and hopelessness. Based on relevant theories and literature, we proposed the following hypotheses and hypothesis model (Figure 1):

*Hypothesis 1*: Perceived social support had positive influence on the mental health of college soccer players.

*Hypothesis 2*: Athlete burnout played a mediating role in the relationship between perceived social support and mental health of college soccer players.

*Hypothesis 3*: Hopelessness played a mediating role in the relationship between perceived social support and mental health of college soccer players.

*Hypothesis 4*: Athlete burnout and hopelessness played a chain mediating role between perceived social support and mental health of college soccer players.

## Materials and methods

### Participants

In this study, the data of college football players in 29 provincial administrative regions, such as Beijing, Shanghai, Chongqing, Guangdong and Shandong, were collected in February, 2022 by the method of convenient sampling. To ensure the validity of the questionnaire, we contacted the head coach of each college sports team, who distributed the questionnaire to the athletes in face-to-face meetings. All participants were informed of the purpose of the study and filled it out voluntarily and anonymously. A total of 695 questionnaires were sent out. After excluding invalid questionnaires, a total of 674 valid questionnaires were obtained with effective recovery of 96.69%. Among them, 418 (62.02%) were male and 256 (37.98%) were female. There were 305 (45.25%) students in rural areas and 369 (54.75%) in urban areas. There were 128 (18.99%) students who have played football for 1–5 years, 399 (59.20%) students for 6–10 years, 116 (17.21%) students for 11–13 years and 31 (4.60%) students for 14 years and more. This study was approved by the Ethics Committee of Shandong University (No. 2021-1-114).

### Subsection

#### Perceived social support

This study adopted the perceived social support scale (PSSS) prepared by Blumenthal et al. and revised by Jiang et al. and Yan et al. (Wang et al., 1999). The scale contained three dimensions of family support, friend support and others support, with a total of 12 items. Likert 7-point scoring method was adopted, with numbers 1–7 representing “strongly disagree” to “strongly agree” respectively, and the total score was the sum of 12 items. The total score between 12 and 36 was low support, between 37 and 60 was medium support, and between 61 and 84 was high support. The higher the total score was, the higher the level of perceived social support was. The Cronbach’s α of the questionnaire in this study was 0.969.

#### Mental health

The mental health was evaluated by Kessler Psychological Distress Scale (K10), which was developed by Kessler et al. and was translated into Chinese by Xu et al. based on K10 Scale (Xu et al., 2005; Kessler et al., 2009). The scale contained 10 items in total, and Likert 5-point scoring method was adopted. Questions were graded 1–5 according to “all the time,” “most of the time,” “some of the time,” “a small part of the time” and “none,” and the score ranged from 10 to 50 points. The higher the score was, the healthier the psychology was. This scale was verified to have good reliability and validity (Zhou et al., 2008). The Cronbach’s α of the questionnaire in this study was 0.969.

#### Athlete burnout

Athlete burnout was measured by the Athlete Burnout Questionnaire (ABQ) developed by Raedeke and Smith and revised by Zhang et al., which contained 15 items in three dimensions: physical/emotional exhaustion, diminished personal accomplishment and negative evaluation of sports (Wang et al., 1999; Zhang and Mao, 2010). The questionnaire used Likert 5-point score, which was “never feel like this” (1 point), “seldom feel like this” (2 points), “sometimes feel like this” (3 points), “often feel like this” (4 points) and “always feel like this” (5 points). The first and 14th topics were reverse scoring, and the total score was the sum of 15 items, with the score ranged from 15 to 75 points. The higher the score was, the higher the athlete burnout of athletes was. The Cronbach’s α of the questionnaire in this study was 0.897.

#### Hopelessness

Beck et al. compiled the Beck Hopelessness Scale (BHS), which contained a total of 20 items from three dimensions: feeling for the future, loss of motivation and expectation for the future (Beck et al., 1974). The Chinese version of this scale was verified with good reliability and validity in Chinese adolescents (Kong et al., 2007). The scale was scored by 0 and 1. Questions 1, 3, 5, 6, 8, 10, 13, 15, and 19 were assigned 0 points for “yes” and 1 point for “no.” The other questions were the opposite. The total score was a sum of 20 items, with a higher score indicating a higher degree of hopelessness. The Cronbach’s α of this scale was 0.744.

### Statistical analysis

In this study, SPSS 26.0 was used for data analysis and Amos 24.0 was used to establish the structural equation model. First, a chain mediating effect test was conducted using PROCESS Macrov3.3 (Hayes, 2013) to assess the indirect effect of perceived social support on mental health through athlete burnout, hopelessness, athlete burnout and hopelessness. PROCESS could automatically generate bootstrap confidence intervals to account for the possible non-normality of the sampling distribution. Then, the overall fit and path analysis of the model were given by Amos. Bootstrap in this study was based on 5,000 samples and generated the 95% confidence interval to test the significance of indirect effect. If the 95% confidence interval did not include 0, it indicated that indirect effect was significant (Preacher and Hayes, 2008).

## Results

### Common method bias test

Since the data in this study were all self-reported by the subjects, there might be significant deviations in the results. Before data analysis, Harman’s single-factor test was used to test whether there was common method bias (Zhou and Long, 2004), and exploratory factor analysis was conducted on all subjects of the study variables. The results showed that there are 9 factors with eigenvalues greater than 1, and the explained variation of the first main factor is 26.57%, lower than the critical value of 40%, indicating that there is no obvious common method bias.

### Descriptive statistics and correlation analysis

Age, gender, place of origin and duration of sports career were used as control variables. Table 1 lists the mean, standard deviation and Pearson correlation coefficients of the main study variables. The results showed that perceived social support was negatively correlated with athlete burnout and hopelessness (*r* = −0.323, *p* < 0.01; *r* = −0.387, *p* < 0.01), and positively correlated with mental health (*r* = 0.390, *p* < 0.01). There was a significant positive correlation between athlete burnout and hopelessness (*r* = 0.317, *p* < 0.01). There was a significant negative correlation between athlete burnout and mental health (*r* = −0.405, *p* < 0.01), and there was also a significant negative correlation between hopelessness and mental health (*r* = −0.461, *p* < 0.01). The relationship between variables supported subsequent testing of the hypothesis.

**Table 1:** Descriptive statistics and Pearson correlation coefficients for main study variables (*n* = 674).

Variables	M	SD	1	2	3	4
1. Perceived Social Support	60.714	14.616	1			
2. Athlete Burnout	34.519	10.255	−0.323^**^	1		
3. Hopelessness	5.497	3.514	−0.387^**^	0.317^**^	1	
4. Mental Health	39.972	9.138	−0.390^**^	−0.405^**^	−0.461^**^	1

M, Mean; SD, Standard deviation.

** *p* < 0.01.

### Chain mediation effect test

Before the chain mediation effect test, this study first examined the direct predictive effect of perceived social support on the mental health of college soccer players. The results showed that the model fitted the data well (x^2^/df = 8.904, CFI = 0.906, TLI = 0.896, RMSEA = 0.108), and perceived social support significantly positively predicted mental health (*B* = 0.247, *p* < 0.01), supporting Hypothesis 1. Then, the chain mediation effect was tested by Model 4 in PROCESS macro. After controlling for gender, age, place of origin and duration of sports career, regression analysis results showed that perceived social support significantly negatively predicted athlete burnout (*B* = −0.227, *p* < 0.01) and hopelessness (*B* = −0.076, *p* < 0.01); athlete burnout positively predicted hopelessness (*B* = 0.075, *p* < 0.01) and negatively predicted mental health (*B* = −0.223, *p* < 0.01); and hopelessness significantly negatively predicted mental health (*B* = −0.772, *p* < 0.01; see Table 2).

**Table 2:** Regression analysis of the relationship between variables (*n* = 674).

	Mental health			Athlete burnout			Hopelessness			Mental health		
Variables	B	SE	t	B	SE	t	B	SE	t	B	SE	t
Constant	30.203^**^	4.486	6.732	46.442^**^	5.218	8.901	6.548^**^	1.791	3.657	48.307^**^	4.315	11.195
Sex	1.872^**^	0.697	2.684	0.426	0.811	0.525	−0.663^*^	0.263	−2.519	1.480^*^	0.631	2.345
Age	−0.373	0.201	−1.860	0.194	0.233	0.831	0.062	0.076	0.821	−0.271	0.181	−1.496
Residence	0.092	0.685	0.134	−0.741	0.797	−0.930	0.308	0.259	1.190	0.121	0.618	0.196
Sports career length	−0.154	0.476	−0.324	−0.785	0.553	−1.419	0.087	0.180	0.484	−0.307	0.429	−0.716
Perceived social support	0.247^**^	0.022	11.095	−0.227^**^	0.026	−8.760	−0.076^**^	0.009	−8.567	0.125^**^	0.022	5.587
Athletic burnout							0.075^**^	0.013	5.950	−0.223^**^	0.031	−7.263
Hopelessness										−0.772^**^	0.092	−8.358
R_2_	0.170			0.108			0.202			0.329		
*F*	27.287^***^			16.197^**^			28.086^**^			46.582^**^		

Gender, age, residence, and sports career length are the control variables, perceived social support is an independent variable, and mental health is a result variable. This table presents the results of the multiple hierarchical regression analysis of perceived social support, athletic burnout, hopelessness, and mental health.

* *p* < 0.05;

** *p* < 0.01;

*** *p* < 0.001.

In order to test the rationality of the chain mediation effect hypothesis, three models were established, namely full mediation model M1, partial mediation model M2 and chain mediation model M3, with perceived social support as the independent variable, mental health as the dependent variable, and athlete burnout and hopelessness as the mediating variables. The fully mediating model M1 was that perceived social support acted on mental health through two mediating variables, namely, athlete burnout and hopelessness. Partial mediation model M2 was based on M1, adding the direct effect path of perceived social support on mental health. The chain mediation model M3 was based on M2, adding the direct effect path of athlete burnout on hopelessness. It can be seen from Table 2 that model M3 is a saturated model, that is, all parameters to be estimated were exactly equal to the elements in the covariance matrix, and the degree of freedom was 0. Therefore, the fitting index was no longer estimated, and only the path coefficient was concerned.

**Figure 2:**
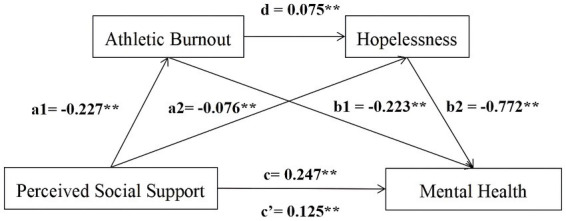
The chain mediation model. The chain mediation model shows the effects of perceived social support, athlete burnout and hopelessness on mental health. *N* = 674. ^**^p < 0.01.

Use the nonparametric percentage Bootstrap program with deviation correction to test the significance of mediation effect, repeat sampling for 5,000 times, and calculate the 95% confidence interval. The direct effect value between perceived social support and mental health was 0.125, and the total effect value was 0.247. This study included three paths of mediation effect. The first path: perceived social support → athlete burnout → mental health, with an indirect effect value of 0.05. The 95% confidence interval was [0.049, 0.119], which did not contain 0, indicating that athlete burnout played a significant mediating role between perceived social support and mental health. Hypothesis 2 was established. The second path: perceived social support → hopelessness → mental health, with an indirect effect value of 0.059. The 95% confidence interval was [0.063, 0.129], which did not contain 0, indicating that hopelessness played a significant mediating role between perceived social support and mental health, and hypothesis 3 was established. The third path: perceived social support → athlete burnout → hopelessness → mental health with an indirect effect value of 0.013. The 95% confidence interval was [0.012, 0.033], also did not include 0, indicating that the chain mediation effect was significant, and hypothesis 4 was established. Thus, athlete burnout and hopelessness were a chain mediator between perceived social support and mental health (see Table 3; Figure 2).

**Table 3:** Results of chain-mediated effect test (*n* = 674).

	Path	Effect	Boot SE	Boot LLCI	Boot ULCI
Direct effect	Perceived social support → mental health	0.125	0.022	0.081	0.168
Indirect effect	Perceived social support → athlete burnout → mental health	0.051	0.017	0.049	0.119
	Perceived social support → hopelessness → mental health	0.059	0.017	0.063	0.129
	Perceived social support → athlete burnout → hopelessness → mental health	0.013	0.005	0.012	0.033
Total effect	Perceived social support → mental health	0.247	0.022	0.203	0.291

LLCI, lower 95% level confidence interval; ULCI, upper 95% level confidence interval.

## Discussion

### The relationship between perceived social support and mental health

The results of this study found that there was a significant positive correlation between perceived social support and mental health of college football players, that was, the higher perceived social support was, the less likely mental health problems would occur, which was consistent with previous results (Calsyn et al., 2005; Ye et al., 2016). Social Support Theory believed that social support could reduce negative effects by guiding people’s interpretation of stressful situations, so as to protect individuals from the negative effects of stressors (Lakey and Cohen, 2000). The stress buffer hypothesis also suggested that high levels of perceived social support could protect individuals from the potential negative effects of stressors (Cohen and Wills, 1985). Chinese college soccer players not only had to complete the training and competition tasks, but also completed the school s academic tasks, and they were under double pressure. At this point, the support of coaches, peers and family members could relieve their anxiety, helplessness and stress, and protected their mental health (Hardy et al., 1991; Noblet et al., 2003). For college athletes who lived away from home, the support and care of coaches were the most important source of social support for athletes, and were the basis for helping athletes effectively cope with pressure and maintain physical and mental health (Freeman and Rees, 2010; Gabana et al., 2017). College athletes relied more on the support of coaches after injury, which could help them overcome fear and anxiety and also had a positive impact on the recovery process (Newman and Weiss, 2018). Therefore, perceived social support of college soccer players is a very important factor for mental health.

### The chain mediating effect of athlete burnout and hopelessness

The results of this study showed that perceived social support can not only directly affected the mental health of college soccer players, but also indirectly affected through three intermediary paths. Firstly, athlete burnout played a mediating role between perceived social support and mental health. The higher perceived social support of college soccer players, the less athlete burnout, and the stronger positive impact on mental health. Athlete burnout is the test of every athlete, and it has a great impact on athletes’ competitive level, training efficiency and physical and mental health. The results of this study were consistent with those of previous studies (Gustafsson et al., 2017; Hartley and Coffee, 2019). According to the conservation of resource theory, individuals should strive to obtain, preserve and maintain the resources with important value to them. When the tasks they faced increase but the resources were depleted, or the resource input and return were out of balance, individuals would feel that resources were threatened or missing. Because they could not adapt to the situation well, they suffered from psychological burnout (Hobfoll, 2001). Athlete burnout was the pressure effect of long-term imbalance between environmental requirements and individual resources (Li and Hou, 2005). The perceived social support of college soccer players was mainly obtained from coaches, peers and family members, which was their important social environment resources. Therefore, the higher their perceived social support, the less athlete burnout they experienced. According to the cognitive-emotional stress model, burnout and stress were parallel development processes, which went through four stages: environmental needs, cognitive evaluation, negative cognitive evaluation and behavioral response. Stress was also the foundation of burnout (Fisher, 1985). In addition, overtraining was also a key factor leading to athlete burnout in athletes (Silva III, 1990). College soccer coaches often required athletes to increase training hours and loads, especially in the early stage of the competition, because they undertook the competition tasks of the school (Cresswell and Eklund, 2005). College soccer players were prone to athlete burnout under overtraining and pressure, which would lead to negative psychological symptoms such as decreased interest, irritability, anxiety and depression, and thus negatively impacting their mental health (Demirci and Çepikkurt, 2018). Therefore, a high level of perceived social support can reduce and prevent athletic burnout in college football players, further reducing mental health problems such as depression and anxiety.

Secondly, the results of this study showed that college soccer players perceived social support could indirectly affect their mental health through hopelessness. The higher the perceived social support of college soccer players, the less their hopelessness and the higher their mental health, which was consistent with previous results (Smith, 1986; Waszczuk et al., 2016; Mac Giollabhui et al., 2017). The hopelessness theory of depression hold that some individuals had cognitive susceptibility to depression. When negative events occurred, individuals were prone to hopelessness, which increased the possibility of depression and was harmful to their mental health. This theory regarded hopelessness as the proximal sufficient cause of the symptoms of hopelessness depression (Smith, 1986). Due to the randomness of soccer matches, college soccer players might feel a strong sense of helplessness and incompetence when they made mistakes or failed to match with the other team members, and they were easy to lose confidence and hopelessness for the following matches or even their future sports career. Athletes suffering from long-term hopelessness might suffer from mental health problems such as depression and suicidal ideation. When the athletes made mistakes or fail in the competition, the coaches, peers or family members should give them sufficient comfort and support after the competition, and rationally analyzed the reasons for the failure to the athletes. This made them not only felt warm and love, but also rationally discovered their shortcomings and made up for them in the later training, so that athletes were less prone to hopelessness. Thus, perceived social support can have an impact on mental health through hopelessness.

This study further revealed that perceived social support could indirectly affect the mental health of college soccer players through the chain mediation of athlete burnout and hopelessness. Hopelessness theory believed that negative life events and negative inference types were the initial and indirect causes of hopeless depression (Smith, 1986). College soccer players had to bear the double pressure of study and competition, and also experienced negative events such as over-training and match failure, so they were easy to appear athlete burnout. Athlete burnout would be accompanied by negative emotions and behaviors such as emotional exhaustion, decreased sense of achievement, lack of sports motivation, and decreased training efficiency (Demirci and Çepikkurt, 2018), and then athletes could not give full play to their best strength in competitions, falling into a negative cycle. Over time this can led to a sense of hopelessness about the future of the sport. Therefore, the athlete burnout of college soccer players has a positive effect on hopelessness, and athlete burnout and hopelessness play a chain mediating role between perceived social support and mental health of Chinese college soccer players.

### Limitations and future research directions

This study had explored the impact of perceived social support on mental health of college soccer players, and deeply analyzed the mediating effects of athlete burnout and hopelessness, which enriched the research fields of perceived social support, athletic burnout, hopelessness, and mental health, and provided empirical support for protecting and promoting the mental health of college soccer players. However, there were some limitations to this study. Firstly, due to time and space constraints, this study adopted a cross-sectional design, which was insufficient to explain the causal relationship between perceived social support and mental health. In the future, experiments or longitudinal studies can be used to further study the causal relationship between variables. Secondly, this study chose Chinese college soccer players as research objects, which is not suitable for other age groups or other sports. Future research could be extended to other age groups and other sports. Finally, although this study adopted the method of face-to-face questionnaire between coaches and athletes, it is difficult to exclude the differences between respondents answers and the actual situation by self-report questionnaire. Therefore, future studies can choose multiple dimensions to collect data.

## Conclusion

This study explored the relationship between perceived social support and mental health of Chinese college soccer players and its mechanism. By constructing a chained mediation model, we found that perceived social support not only directly predicted mental health, but also indirectly through the separate mediating effect of athlete burnout and hopelessness. In addition, it can indirectly predict mental health through the chain mediation effect of athlete burnout and hopelessness.

## Data availability statement

The raw data supporting the conclusions of this article will be made available by the authors, without undue reservation.

## Ethics statement

The studies involving human participants were reviewed and approved by the Ethics Committee of Shandong University (No. 2021-1-114). Written informed consent to participate in this study was provided by the participants’ legal guardian/next of kin.

## Author contributions

LZhang: methodology, validation, investigation, resources, data curation, writing—review and editing, supervision, project administration, and funding acquisition. LZhao: conceptualization, methodology, software, investigation, writing—original draft preparation, and writing—review and editing. ZL: conceptualization, formal analysis, and visualization. All authors contributed to the article and approved the submitted version.

## Funding

This research was funded by Shandong Humanities and Social Sciences Project, grant number 20CLYJ34.

## Conflict of interest

The authors declare that the research was conducted in the absence of any commercial or financial relationships that could be construed as a potential conflict of interest.

## Publisher’s note

All claims expressed in this article are solely those of the authors and do not necessarily represent those of their affiliated organizations, or those of the publisher, the editors and the reviewers. Any product that may be evaluated in this article, or claim that may be made by its manufacturer, is not guaranteed or endorsed by the publisher.

## References

[ref1] Abbott W. Brownlee T. E. Harper L. D. Naughton R. J. Richardson A. Clifford T. (2019). A season long investigation into the effects of injury, match selection and training load on mental wellbeing in professional under 23 soccer players: a team case study. Eur. J. Sport Sci. 19, 1250–1256. doi: 10.1080/17461391.2019.1600586, PMID: 30955458

[ref2] Abramson L. Y. Metalsky G. I. Alloy L. B. (1989). Hopelessness depression: a theory-based subtype of depression. Psychol. Rev. 96, 358–372. doi: 10.1037/0033-295X.96.2.358

[ref3] Akesdotter C. Kentta G. Eloranta S. Franck J. (2020). The prevalence of mental health problems in elite athletes. J. Sci. Med. Sport 23, 329–335. doi: 10.1016/j.jsams.2019.10.022 31806359

[ref4] Allen H. K. Lilly F. Green K. M. Zanjani F. Vincent K. B. Arria A. M. (2020). Graduate student burnout: substance use, mental health, and the moderating role of advisor satisfaction. Int. J. Ment. Health Ad. 20, 1130–1146. doi: 10.1007/s11469-020-00431-9, PMID: 35400127 PMC8992873

[ref5] Au R. C. Watkins D. Hattie J. Alexander P. (2009). Reformulating the depression model of learned hopelessness for academic outcomes. Educ. Res. Rev. 4, 103–117. doi: 10.1016/j.edurev.2009.04.001

[ref6] Baryshnikov I. Rosenstrom T. Jylha P. Koivisto M. Mantere O. Suominen K.. (2008). State and trait hopelessness in a prospective five-year study of patients with depressive disorders. J. Affect. 239, 107–114. doi: 10.1016/j.jad.2018.07.007, PMID: 29990656

[ref7] Beck A. T. Weissman A. Lester D. Trexler L. (1974). Measurement of pessimism: hopelessness scale. J. Consult. Clin. Psychol. 42, 861–865. doi: 10.1037/h0037562, PMID: 4436473

[ref9] Calsyn R. J. Winter J. P. Burger G. K. (2005). The relationship between social anxiety and social support in adolescents: a test of competing causal models. Adolescents 40, 103–113. 15861620

[ref1006] Cheng L. Zhang Z. (2014). Development and Enlightenment of Japanese Football Youth Training. Sports Culture Guide. 7, 95–98.

[ref10] Chengchao Z. Jie C. Ting W. Qianqian P. Jiangjiang H. Wengui Z.. (2008). Evaluation of the reliability and validity of the Chinese version of Kessler 10, a simple mental state rating scale. Chin. J. Clin. Psych. 16, 627–629.

[ref11] China Youth Daily. (2022). Available at: https://baijiahao.baidu.com/s?id=1690549240307735204&wfr=spider&for=pc (Accessed 15 March 2022).

[ref12] Cohen S. Wills T. A. (1985). Stress, social support, and the buffering hypothesis. Psychol. Bull. 98, 310–357. doi: 10.1037/0033-2909.98.2.310 3901065

[ref13] Cresswell S. L. (2009). Possible early signs of athlete burnout: a prospective study. J. Sci. Med. Sport 12, 393–398. doi: 10.1016/j.jsams.2008.01.009, PMID: 18356101

[ref14] Cresswell S. L. Eklund R. C. (2005). Changes in athlete burnout and motivation over a 12-week league tournament. Med. Sci. Sports Exerc. 37, 1957–1966. doi: 10.1249/01.mss.0000176304.14675.32, PMID: 16286867

[ref15] DeFreese J. D. Smith A. L. (2014). Athlete social support, negative social interactions, and psychological health across a competitive sport season. J. Sport Exerc. Psychol. 36, 619–630. doi: 10.1123/jsep.2014-0040, PMID: 25602144

[ref16] Demirci E. Çepikkurt F. (2018). Examination of the relationship between passion, perfectionism and burnout in athletes. Univ. J. Educ. Res. 6, 1252–1259. doi: 10.13189/ujer.2018.060616

[ref17] Doherty S. Hannigan B. Campbell M. J. (2016). The experience of depression during the careers of elite male athletes. Front. Psychol. 7:1069. doi: 10.3389/fpsyg.2016.01069, PMID: 27486418 PMC4947597

[ref18] Dunkel-Schetter C. Bennett T. L. (1990). “Differentiating the cognitive and behavioral aspects of social support,” in Social support: An interactional view. eds. Sarason B. R. Sarason I. G. Pierce G. R. (New York: Wiley), 267–296.

[ref19] Elbe A. M. Jensen S. N. (2016). Commentary: comparison of athletes’ proneness to depressive symptoms in individual and team sports: research on psychological mediators in junior elite athletes. Front. Psychol. 7:1782. doi: 10.3389/fpsyg.2016.01782, PMID: 27917134 PMC5114271

[ref20] Fan F. C. Zhang S. Y. Cheng Y. (2021). Incidence of psychological illness after coronavirus outbreak: a meta-analysis study. J. Epidemiol. Community Health 75, 836–842. doi: 10.1136/jech-2020215927, PMID: 33632722 PMC7908057

[ref21] Fanglin L. Liwei Z. (2004). Qualitative exploration of Athletes’ psychological fatigue. China Sport Sci. 11, 37–44. doi: 10.16469/j.css.2004.11.010

[ref22] Fisher C. D. (1985). Social support and adjustment to work: a longitudinal study. J. Manage. 11, 39–53. doi: 10.1177/014920638501100304

[ref23] Fletcher D. Sarkar M. (2012). A grounded theory of psychological resilience in Olympic champions. Psychol. Sport Exerc. 13, 669–678. doi: 10.1016/j.psychsport.2012.04.007

[ref24] Freeman P. Rees T. (2008). The effects of perceived and received support upon objective performance outcome. Eur. J. Sport Sci. 8, 359–368. doi: 10.1080/17461390802261439

[ref25] Freeman P. Rees T. (2010). Perceived social support from team-mates: direct and stress-buffering effects on self-confidence. Eur. J. Sport Sci. 10, 59–67. doi: 10.1080/17461390903049998

[ref26] Freudenberger H. J. (1974). Staff burn-out. JSI 30, 159–165. doi: 10.1111/j.1540-4560.1974.tb00706.x

[ref27] Gabana N. T. Steinfeldt J. A. Wong Y. J. Chung Y. B. (2017). Gratitude, burnout, and sport satisfaction among college student-athletes: the mediating role of perceived social support. J. Clin. Sport Psychol. 11, 14–33. doi: 10.1123/jcsp.2016-0011

[ref28] Gerber M. Lang C. Feldmeth A. K. Elliot C. Brand S. Holsboer-Trachsler E.. (2015). Burnout and mental health in Swiss vocational students: the moderating role of physical activity. J. Res. Adolesc. 25, 63–74. doi: 10.1111/jora.12097

[ref29] Guney S. Kalafat T. Boysan M. (2010). Dimensions of mental health: life satisfaction, anxiety and depression: a preventive mental health study in Ankara University students population. Innov. Creat. Educ. 2, 1210–1213. doi: 10.1016/j.sbspro.2010.03.174

[ref31] Gustafsson H. DeFreese J. D. Madigan D. J. (2017). Athlete burnout: review and recommendations. Curr. Opin. Psychol. 16, 109–113. doi: 10.1016/j.copsyc.2017.05.002, PMID: 28813331

[ref32] Gustafsson H. Hassmén P. Podlog L. (2010). Exploring the relationship between hope and burnout in competitive sport. J. Sports Sci. 28:1504. doi: 10.1080/02640414.2010.521943 21077003

[ref33] Hardy C. J. Richman J. M. Rosenfeld L. B. (1991). The social support survey - a validation-study of a clinical measure of the social support process. Sport Psychol. 3, 288–311. doi: 10.1177/104973159300300304

[ref34] Hartley C. Coffee P. (2019). Perceived and received dimensional support: Main and stress-buffering effects on dimensions of burnout. Front. Psychol. 10:1724. doi: 10.3389/fpsyg.2019.01724, PMID: 31428013 PMC6687870

[ref35] Hayes A. F. (2013). An introduction to mediation, moderation, and conditional process analysis: A regression-based approach. USA: The Guilford Press.

[ref36] Heerde J. A. Hemphill S. A. (2018). Examination of associations between informal help-seeking behavior, social support, and adolescent psychosocial outcomes: a meta-analysis. Dev. Rev. 47, 44–62. doi: 10.1016/j.dr.2017.10.001

[ref37] Henriksen K. Schinke R. Moesch K. McCann S. Parham W. D. Larsen C. H.. (2019). Consensus statement on improving the mental health of high performance athletes. Int. J. Sport Psychol. 18, 553–560. doi: 10.1080/1612197X.2019.1570473

[ref38] Hobfoll S. E. (2001). The influence of culture, community, and the nested-self in the stress process: advancing conservation of resources theory. Appl. Psychpl. Int. Rev. Psychpl. Appl. Rev. Int. 50, 337–421. doi: 10.1111/1464-0597.00062

[ref39] Hobfoll S. E. Tirone V. Holmgreen L. Gerhart J. (2016). “Conservation of resources theory applied to major stress,” in stress concepts and cognition, emotion, and behavior (London: Elsevier Inc.)

[ref40] Jakobsen A. L. Hansen C. D. Andersen J. H. (2020). The association between perceived social support in adolescence and positive mental health outcomes in early adulthood: a prospective cohort study. Scand. J. Public Health 50, 404–411. doi: 10.1177/1403494821993718, PMID: 33645305

[ref41] Johnston L. H. Carroll D. (1998). The provision of social support to injured athletes: a qualitative analysis. J. Sport Rehabil. 7, 267–284. doi: 10.1123/jsr.7.4.267

[ref42] Kahill S. (1988). Symptoms of professional burnout: a review of the empirical evidence. Can. Psychol. 29, 284–297. doi: 10.1037/h0079772

[ref43] Kessler R. C. Aguilar-Gaxiola S. Wang P. S. (2009). The global burden of mental disorders: an update from the WHO world mental health (WMH) surveys. Epidemiol. Psichiatr. Soc. 18, 23–33. doi: 10.1017/S1121189X00001421, PMID: 19378696 PMC3039289

[ref1008] Kong Y. Y. Zhang J. Jia S. H. Zhou L. (2007). Reliability and validity of Chinese version of Beck Despair Scale in adolescents. Chinese Mental Health Journal. 21, 686–689. doi: 10.3321/j.issn:1000-6729.2007.10.008

[ref44] Lakey B. Cohen S. (2000). “Social support theory and measurement,” in Social support measurement and intervention: A guide for health and social scientists. eds. Cohen S. Underwood L. G. Gootlieb B. H. (Oxford: Oxford University Press).

[ref45] Lamis D. A. Ballard E. D. May A. M. Dvorak R. D. (2016). Depressive symptoms and suicidal ideation in college students: the mediating and moderating roles of hopelessness, alcohol problems, and social support. J. Clin. Psychol. 72, 919–932. doi: 10.1002/jclp.22295, PMID: 27008096

[ref46] Lester D. (2015). Hopelessness in adolescents. J. Affect. Disord. 173, 221–225. doi: 10.1016/j.jad.2014.10.048 25462420

[ref47] Li Y. (2014). Relationship among hopelessness, resilience and suicidal ideation of college students. Chongqing Med. J. 43, 524–526. doi: 10.3969/j.issn.1671-8348.2014.05.005

[ref1007] Li Y. X. Hou Y. (2005). Burnout, stress and depressionz. Psychological Science. 28, 972–974. doi: 10.16719/j.cnki.1671-6981.2005.04.053

[ref48] Lingzhong X. Jianxin W. Hui S. Xiyu Z. Xingzhou W. Chengchao Z.. (2005). The first application of Kessler 10 in China and its significance. Soft Sci. Health 19, 410–412.

[ref1001] Liu B. (2011). Enlightenment of German Sports System Research on Further Perfecting China’s Sports System. Journal of Beijing Sport University. 34:5-9+14. doi: 10.19582/j.cnki.11-3785/g8.2011.11.002

[ref51] Lv Y. Bin W. Min W. Zunjia L. Liangshan D. (2016). The effect of social support on athlete engagement: the serial mediating role of psychological toughness and coping style. J. Beijing Sport Univ. 39, 75–82. doi: 10.19582/j.cnki.11-3785/g8.2016.07.011

[ref52] Mac Giollabhui N. Hamilton J. L. Nielsen J. Connolly S. L. Stange J. P. Varga S.. (2017). Negative cognitive style interacts with negative life events to predict first onset of a major depressive episode in adolescence via hopelessness. J. Abnorm. Psychol. 127, 1–11. doi: 10.1037/abn0000301, PMID: 29172599 PMC5785411

[ref1102] Ma G. (2016). The relationship between rumination and depression: A mediating model of cognitive fusion. master’ s thesis. China: Harbin Engineering University.

[ref53] Maslach C. Jackson S. E. (1982). “Burnout in health professions: a social psychological analysis,” in Social Psychology of Health and Illness. Hillsdale, NJ: Lawrence Erlbaum. 227–251.

[ref54] Nassar M. F. Allam M. F. Shata M. O. (2021). Effect of COVID-19 lockdown on young Egyptian soccer players. GPH 8:2333794X211012980. doi: 10.1177/2333794X211012980, PMID: 34017905 PMC8114258

[ref55] Newman N. D. Weiss W. M. (2018). Relationship between demographic variables and collegiate athletes' perceptions of social support from head coaches. Int. J. Sports Sci. Coach. 13, 343–348. doi: 10.1177/1747954117737985

[ref56] Noblet A. Rodwell J. McWilliams J. (2003). Predictors of the strain experienced by professional Australian footballers. J. Appl. Sport Psychol. 15, 184–193. doi: 10.1080/10413200305394

[ref57] Pekrun R. Elliot A. J. Maier M. A. (2009). Achievement goals and achievement emotions: testing a model of their joint relations with academic performance. J. Educ. Psychol. 101, 115–135. doi: 10.1037/a0013383

[ref58] Pompili M. Rinaldi G. Lester D. Girardi P. Ruberto A. Tatarelli R. (2006). Hopelessness and suicide risk emerge in psychiatric nurses suffering from burnout and using specific defense mechanisms. Arch. Psychiatr. Nurs. 20, 135–143. doi: 10.1016/j.apnu.2005.12.002, PMID: 16716857

[ref59] Preacher K. J. Hayes A. F. (2008). Asymptotic and resampling strategies for assessing and comparing indirect effects in multiple mediator models. Behav. Res. Methods 40, 879–891. doi: 10.3758/brm.40.3.879, PMID: 18697684

[ref1101] Qiu T. (2015). The mediating effect of perceived social support on performance of basketball players under stress.. J. Wuhan Instit. Phys. Educ. 5, 96–100. doi: 10.15930/j.cnki.wtxb.2015.04.018

[ref60] Raedeke T. D. (1997). Is athlete burnout more than just stress? A sport commitment perspective. J. Sport Exercise Psy. 19, 396–417. doi: 10.1123/jsep.19.4.396

[ref61] Raedeke T. D. Smith A. L. (2001). Development and preliminary validation of an athlete burnout measure. J. Sport Exercise Psy. 23, 281–306. doi: 10.1123/jsep.23.4.281, PMID: 28682196

[ref62] Reardon C. L. Hainline B. Aron C. M. Baron D. Baum A. L. Bindra A.. (2019). Mental health in elite athletes: International Olympic Committee consensus statement. Br. J. Sports Med. 53, 667–699. doi: 10.1136/bjsports-2019-100715, PMID: 31097450

[ref63] Rees T. Hardy L. (2000). An investigation of the social support experiences of high-level sports performers. Sport Psychol. 14, 327–347. doi: 10.1123/tsp.14.4.327

[ref64] Roberts C. Faull A. L. Tod D. (2016). Blurred lines: performance enhancement, common mental disorders and referral in the UK. Athletic population. Front. Psychol. 7, 1–13. doi: 10.3389/fpsyg.2016.01067, PMID: 27468273 PMC4942456

[ref65] Salmela-Aro K. Savolainen H. Holopainen L. (2009). Depressive symptoms and school burnout during adolescence: evidence from two cross-lagged longitudinal studies. J. Youth Adolesc. 38, 1316–1327. doi: 10.1007/s10964-008-9334-3, PMID: 19779808

[ref66] Schinke R. Papaioannou A. Henriksen K. Si G. Zhang L. Haberl P. (2020). Sport psychology services to high performance athletes during COVID-19. Int. J. Sport Psychol. 18, 269–272. doi: 10.1080/1612197X.2020.1754616

[ref67] Schinke R. J. Stambulova N. B. Si G. Moore Z. (2018). International society of sport psychology position stand: Athletes’ mental health, performance, and development. Int. J. Sport Psychol. 16, 622–639. doi: 10.1080/1612197X.2017.1295557

[ref68] Scotland E. (1969). The psychology of hope. San Francisco: Jossey-Bass.

[ref69] Serafini G. Lamis D. A. Aguglia A. Amerio A. Nebbia J. Geoffroy P. A.. (2020). Hopelessness and its correlates with clinical outcomes in an outpatient setting. J. Affect. 263, 472–479. doi: 10.1016/j.jad.2019.11.144, PMID: 31969280

[ref70] Sheridan D. Coffee P. Lavallee D. (2014). A systematic review of social support in youth sport. Int. Rev. Sport Exerc. Psychol. 7, 198–228. doi: 10.1080/1750984X.2014.931999

[ref71] Silva J. M. III (1990). An analysis of the training stress syndrome in comp etitive athletics. J. Appl. Sport Psychol. 2, 5–20. doi: 10.1080/10413209008406417

[ref72] Smith R. E. (1986). Toward a cognitive - affective model of athletic burnout. J. Sport Psychol. 8, 36–50. doi: 10.1123/jsp.8.1.36

[ref73] Sun S. H. Zhang S. X. Jahanshahi A. A. Jahanshahi M. (2021). Drilling under the COVID-19 pandemic: a diary study of professional football players' mental health and workout performance. Stress. Health 38, 3–18. doi: 10.1002/smi.3059, PMID: 33945206 PMC8237014

[ref75] Uchino B. N. (2009). Understanding the links between social support and physical health: a life- span perspective with emphasis on the separability of perceived and received support. Perspect. Psychol. Sci. 4, 236–255. doi: 10.1111/j.1745-6924.2009.01122.x, PMID: 26158961

[ref76] Uchino B. N. Cacioppo J. T. Kiecolt-Glaser J. K. (1996). The relationship between social support and physiological processes: a review with emphasis on underlying mechanisms and implications for health. Psychol. Bull. 119, 488–531. doi: 10.1037/0033-2909.119.3.488, PMID: 8668748

[ref79] Wang X. Xilin W. Hong M. (1999). Handbook of psychological assessment scale (Revised Edition). Beijing: Chinese Mental Health Journal.

[ref77] Waszczuk M. A. Coulson A. E. Gregory A. M. Eley T. C. (2016). A longitudinal twin and sibling study of the hopelessness theory of depression in adolescence and young adulthood. Psychol. Med. 46, 1935–1949. doi: 10.1017/S0033291716000489, PMID: 27019371

[ref78] World Health Organization. (2018). Mental health: a state of well-being. Available at: http://www.who.int/features/factfiles/mentalhealth/zh/ (Accessed 15 March 2022).

[ref1004] Xu L. Z. Wang J. X. Sun H. Zhang X. Y. Wang X. Z. Zhou C. C.. (2005). The first application of Kessler 10 in China and its significance. Soft Science of Health. 19, 410–412. doi: 10.3969/j.issn.1003-2800.2005.06.013

[ref80] Ye L. Wang B. Ge Y. Bu S. Wang L. (2016). The effect of athletes’ gratitude on burnout—chain mediating effects of social support and mental toughness. China Sport Sci. 36, 39–49. doi: 10.16469/j.css.201611005

[ref81] Yongxin L. Hui H. (2005). Burnout, stress and depressionz. Psychol. Sci. 28, 972–974.

[ref82] Yuanyuan K. Jie Z. Shuhua J. Li Z. (2007). Reliability and validity of Chinese version of Beck despair scale in adolescents. Chin. Ment. Health J. 21, 686–689.

[ref1103] Zhang L. Mao Z. (2010). Evaluation manual of common psychological scales for physical education. Beijing: Beijing Sport University Press.

[ref1003] Zhou C. C. Chu J. Wang T. Peng Q. Q. He J. J. Zheng W. G.. (2008). Evaluation of the reliability and validity of the Chinese version of Kessler10, a simple mental state rating scale. Chinese Journal of Clinical Psychology. 16, 627–629. https://www.webofscience.com/wos/alldb/full-record/CSCD:3457129

[ref83] Zhou H. Long L. R. (2004). Statistical remedies for common method biases. Adv. Psychol. Sci. 12, 942–950. doi: 10.1037/0021-9010.88.5.879

